# Macroinvertebrate Taxonomic and Functional Trait Compositions within Lotic Habitats Affected By River Restoration Practices

**DOI:** 10.1007/s00267-017-0889-1

**Published:** 2017-05-17

**Authors:** J. C. White, M. J. Hill, M. A. Bickerton, P. J. Wood

**Affiliations:** 1 Centre for Hydrological and Ecosystem Science, Department of Geography, Loughborough University, Loughborough, Leicestershire, LE11 3TU UK; 2 Institute of Science and the Environment, University of Worcester, Henwick Grove, Worcester, WR2 6AJ UK; 3 School of Geography, Earth and Environmental Sciences, University of Birmingham, Edgbaston, Birmingham, B15 2TT UK

**Keywords:** Habitat enhancement, Invertebrates, Lotic ecosystems, River rehabilitation, Traits

## Abstract

The widespread degradation of lotic ecosystems has prompted extensive river restoration efforts globally, but many studies have reported modest ecological responses to rehabilitation practices. The functional properties of biotic communities are rarely examined within post-project appraisals, which would provide more ecological information underpinning ecosystem responses to restoration practices and potentially pinpoint project limitations. This study examines macroinvertebrate community responses to three projects which aimed to physically restore channel morphologies. Taxonomic and functional trait compositions supported by widely occurring lotic habitats (biotopes) were examined across paired restored and non-restored (control) reaches. The multivariate location (average community composition) of taxonomic and functional trait compositions differed marginally between control and restored reaches. However, changes in the amount of multivariate dispersion were more robust and indicated greater ecological heterogeneity within restored reaches, particularly when considering functional trait compositions. Organic biotopes (macrophyte stands and macroalgae) occurred widely across all study sites and supported a high alpha (within-habitat) taxonomic diversity compared to mineralogical biotopes (sand and gravel patches), which were characteristic of restored reaches. However, mineralogical biotopes possessed a higher beta (between-habitat) functional diversity, although this was less pronounced for taxonomic compositions. This study demonstrates that examining the functional and structural properties of taxa across distinct biotopes can provide a greater understanding of biotic responses to river restoration works. Such information could be used to better understand the ecological implications of rehabilitation practices and guide more effective management strategies.

## Introduction

A significant number of river restoration projects aiming to rehabilitate degraded lotic ecosystems have been carried out globally (Ormerod 2004; Bernhardt et al. 2007; Miller et al. 2010; Kail et al. 2015). River restoration practices regularly involve changes to the physical template of fluvial environments, with project aims often centered on promoting a range of habitats capable of supporting heterogeneous biotic assemblages (Palmer et al. 2010). However, evidence from a plethora of studies has highlighted that reinstating a greater degree of habitat heterogeneity in lotic environments does not guarantee ecological recovery (e.g., Roni et al. 2008; Miller et al. 2010; Barnes et al. 2013).

Limited ecological responses to river restoration works have been attributed to a multitude of reasons, including socio-economic constraints (Langford and Shaw 2014) and inappropriate spatial scaling of projects (Miller et al. 2010). In addition, restoration schemes are regularly undertaken without the guidance of ecological baseline data, with biomonitoring information often not being collected before or after project implementation (Bernhardt et al. 2007; Kail et al. 2015). Furthermore, various studies have attributed ecologically ineffective rehabilitation projects to prevailing abiotic constraints (e.g., degraded water quality, modified flow regimes) and/or organism dispersal limitations (e.g., Lepori et al. 2005; Jähnig et al. 2010; Tonkin et al. 2014). Palmer et al. (2010) reviewed 78 river restoration projects globally and found no association between habitat heterogeneity and the richness of macroinvertebrate taxa, suggesting that existing restoration techniques have been inappropriate in facilitating ecological recovery. However, quantifying changes in taxonomic richness after restoration works may not necessarily be a suitable biological end point (besides being consistently reported within post-project appraisals) and the need to report functional responses to river restoration efforts is being increasingly advocated (e.g., Dolédec et al. 2015; Kail et al. 2015).

The examination of functional traits (the biological properties and ecological preferences of organisms) is often overlooked within river restoration post-project appraisals (Kail et al. 2015). Processing such information alongside traditional taxonomic-based approaches and quantifying biotic differences between restored and non-restored (control) sites within univariate and multivariate contexts enhances the amount of ecological information available from post-project appraisals. Univariate taxonomic responses allow target organisms (e.g., non-native taxa) to be examined, while individual traits may infer causal mechanisms underpinning biotic responses to river restoration practices by highlighting the sensitivity of specific faunal properties (e.g., Jähnig and Lorenz 2008; Tullos et al. 2009). Multivariate analysis of taxonomic and trait compositions allows different types of community responses to be examined. Within lotic environments, shifts in the multivariate location (the average community composition) of biotic communities are often the result of large-scale environmental variables which have similar environmental implications across an entire river catchment or region (Poff 1997). However, the amount of multivariate dispersion is likely to be more sensitive to small-scale variables which have localized biotic implications, such as the presence of different habitats between study sites (see Heino et al. 2012).

Lotic “biotopes” comprise various types of mineralogical coverings (e.g., gravel and sand substrate patches) and organic habitats (e.g., macroalgae and macrophyte stands) which arise through hydrological, hydraulic, and geomorphological forces (Armitage et al. 1995; Storey and Lynas 2007). The term “biotopes” (*sensu* Demars et al. 2012) is used in this study as an ecological equivalent to “microhabitats” (*sensu* Frissell et al. 1986), “mesohabitats” (*sensu* Tickner et al. 2000) or “functional habitats” (*sensu* Harper et al. 1998). Various studies have reported that biotopes support distinct faunal compositions (e.g., Harper et al. 1992, 1998; Buffagni et al. 2000; Storey and Lynas 2007) and the recognition of such habitats have underpinned river conservation strategies (Tickner et al. 2000; Harvey and Clifford 2008). In addition, they have been used to appraise biotic responses to different anthropogenic influences within lotic environments including flow regulation (Armitage and Pardo 1995; Storey and Lynas 2007) and river restoration practices (Verdonschot et al. 2016). However, biotope controls on the functional composition of macroinvertebrate communities have not been widely explored within lotic ecosystems, but could offer further ecological information which could underpin river management strategies (see Demars et al. 2012).

This study examines macroinvertebrate taxonomic and functional trait responses to three river restoration projects conducted within the River Tame (East and West Midlands, UK), a historically polluted and physically modified catchment. The restoration techniques used on the projects examined in this study are novel within the UK as they involved fashioning multi-channel systems, which has rarely been incorporated within other restoration projects implemented nationwide (see River Restoration Centre 2013). The study aims are to: (i) quantify differences in macroinvertebrate taxonomic and functional trait compositions between control and restored reaches; (ii) examine if the structural and functional attributes of macroinvertebrate communities differ between distinct biotopes supported by control and restored reaches and (iii) evaluate the advantages of utilizing macroinvertebrate functional traits within river restoration post-project appraisals.

## Methods

### Study Area and Sites

The River Tame represents one of the most urbanized fluvial landscapes in the UK (Webster et al. 2001). Watercourses within the catchment have been historically subjected to heavy metal and nutrient pollution, in addition to widespread morphological changes (Beavan et al. 2001; Langford et al. 2010—see Supplementary Material for water quality parameters). Prior to anthropogenic influences, the Tame exhibited a braided planform as a result of its sandstone lithology (Ellis et al. 2007), but is now characterized by a single-thread morphology throughout the catchment.

**Fig. 1:**
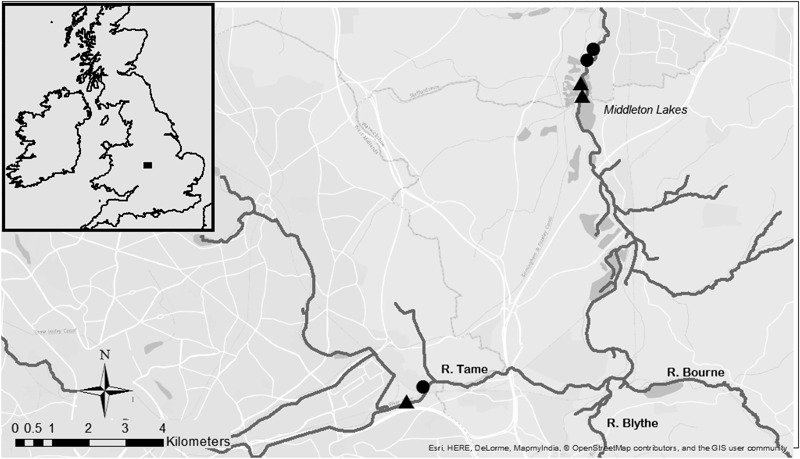
Study sites across the River Tame. *Square* = study location, *Triangles* = control sites, and *Circles* = restored sites

Three restoration schemes were examined which involved reach-scale channel manipulations (project lengths ranging from 253–500 m) aiming to reinstate a multi-channel planform by widening channels and creating mature vegetated islands that divert flows into separate channels (see Supplementary Material for further information on each project). Restoration works were completed between 7 and 16 years prior to fieldwork being conducted in summer 2014, thus allowing sufficient time for the recolonization and recovery of macroinvertebrate communities. Two of the restoration projects were conducted along adjacent stretches of river (see Fig. 1), but were completed 8 years apart and involved different techniques (with one project also creating mid-channel bars which become inundated at higher flows—see Supplementary Material), so were treated as separate sites in this study. With no pre-restoration data being available, a space-time substitution was adopted and each restored reach was paired with a respective non-restored (control) reach situated <1 km upstream. While it is recognized that taxa drifting between sites may have influenced biotic differences between control and restored reaches, sampling faunal assemblages from specific habitat units (see below) would provide fundamental information on whether macroinvertebrates colonized distinct biotopes influenced by river restoration practices As a result of widespread historic anthropogenic modifications throughout the River Tame’s course, no baseline reference conditions or analogs were available to provide a benchmark for restoration outcomes.

### Observations of Biotopes

Biotopes were pre-defined as distinct in-channel habitat patches that were visually distinguishable from the river bank (Armitage et al. 1995; Demars et al. 2012). Preliminary walk-over surveys were conducted to characterize the visually “dominant biotopes” evident within each of the six study reaches, defined herein as those possessing at least three distinct patches (to guide subsequent macroinvertebrate sampling—see below) which collectively dominate the wetted area of the channel (Storey and Lynas 2007). The spatial configuration and prevalence of dominant biotopes were visually mapped onto a river channel outline obtained from Google Earth (2015). This was conducted across the entirety of each study reach to ensure all dominant biotopes were characterized. Mapping the composition of dominant biotopes was not used to quantify habitat heterogeneity per se, but facilitated the identification of widely occurring habitat patches which are artifacts of hydraulic and geomorphological forces (Kemp et al. 1999) influenced by the restoration activities examined in this study. Rare biotopes (including silt patches and woody debris) were not included in the final analyses due to insufficient numbers of replicate samples. Subsequently, six dominant biotopes were identified for sampling, with macroalgae, “*Ranunculus*. sp” (a fine-leaved, submerged macrophyte) and “*Sparganium emersum*” (a broad-leaved, submerged macrophyte) representing the three dominant organic biotopes. Gravel, sand and a mixture of these substrates (whereby gravel clasts were present within a sand matrix) were the three dominant mineralogical biotopes surveyed.

### Macroinvertebrate Sampling

Macroinvertebrates were collected using 15-s kick samples from separate patches of all dominant biotopes observed within each study reach, with three replicate samples being taken (*sensu* Tickner et al. 2000). A total of 69 samples were collected, with a smaller number (*n* = 24) being collected from control reaches due to a reduced number of dominant biotopes being present compared to restored reaches (*n* = 45). Samples were preserved in 70% ethanol in the field and identified to family-level in the laboratory, with the exception of Oligochaeta which were identified as such.

### Functional Traits

Macroinvertebrate functional traits in this study were derived from a database initially developed in France, but which is applicable to other European freshwater systems (Usseglio-Polatera et al. 2000; Tachet et al. 2010). For comparability with other studies, the nomenclature of functional traits is reported herein by their “grouping features” and “traits” (as outlined by Schmera et al. 2015). Grouping features represent a functional trait category (e.g., “life-cycle duration” and “maximum body size”), while traits represent the modalities residing within these (e.g., life-cycle duration—“≤1 year”, “>1 year”; maximum body size—“≤0.25 cm”, “≥8 cm”). The functional trait database employs a “fuzzy-coding” approach, whereby macroinvertebrate affinities for individual traits range from zero (indicating no affinity) to three or five (indicating strong affinity—the maximum value depending on the level of information available in existing literature—see Chevene et al. 1994; Tachet et al. 2010). Information within the original traits database is typically available at species- or genus-level and the processing of traits for use within this research involved a series of steps: (i) removing non-UK taxa (guided by Davies and Edwards 2011) from the initial database (*sensu* Demars et al. 2012), as well as those not observed within this study; (ii) standardizing each grouping feature so that traits summed to 1 (thus ensuring equal taxonomic weighting); (iii) averaging values to match the taxonomic resolution of faunal information available within this study and then standardizing (as above) to account for taxa expressing zero affinity scores across all traits within a specific grouping feature; (iv) multiplying these values by ln(x + 1) transformed community abundances (see Schmera et al. 2014) to create a trait-abundance array; (v) averaging each trait across all sampled taxa and standardizing (as above) to account for between reach abundances (Gayraud et al. 2003; Demars et al. 2012). Thirteen grouping features were analyzed (Table 1), with only two characterizing ecological preferences (velocity and substrate) which were selected a priori due to river widening at restored sites modifying the hydraulic conditions and substrate composition of the channel (as opposed to other ecological preferences within the database—see Tachet et al. 2010).

**Table 1:** Macroinvertebrate functional traits examined within this study

Grouping feature	Trait	Code	Grouping feature	Trait	Code
Maximum potential size	≤0.25 cm	Size.1	Locomotion and substrate relation	Flier	Locomotion.1
	>0.25–0.5 cm	Size.2		Surface swimmer	Locomotion.2
	>0.5–1 cm	Size.3		Full water swimmer	Locomotion.3
	>1–2 cm	Size.4		Crawler	Locomotion.4
	>2–4 cm	Size.5		Burrower	Locomotion.5
	>4–8 cm	Size.6		Interstitial	Locomotion.6
	>8 cm	Size.7		Temporarily attached	Locomotion.7
Life-cycle duration	≤1 year	Life-cycle.1		Permanently attached	Locomotion.8
	>1 year	Life-cycle.2	Food consumed	Microorganisms	Food.1
Voltinism	<1	Voltinism.1		Detritus <1 mm	Food.2
	1	Voltinism.2		Dead plant ≥1 mm	Food.3
	>1	Voltinism.3		Living microphytes	Food.4
Aquatic stages	Egg	Stage.1		Living macrophtyes	Food.5
	Larva	Stage.2		Dead animal ≥1 mm	Food.6
	Nymph	Stage.3		Living microinvertebrates	Food.7
	Adult	Stage.4		Living macroinvertebrates	Food.8
Reproduction strategy	Ovoviviparity	Reproduction.1		Vertebrates	Food.9
	Isolated, free eggs	Reproduction.2	Feeding group	Absorber	Feeding.1
	Isolated, cemented eggs	Reproduction.3		Deposit feeder	Feeding.2
	Clutches, cemented	Reproduction.4		Shredder	Feeding.3
	Clutches, free	Reproduction.5		Scraper	Feeding.4
	Clutches, in vegetation	Reproduction.6		Filter-feeder	Feeding.5
	Clutches, terrestrial	Reproduction.7		Piercer	Feeding.6
	Asexual	Reproduction.8		Predator	Feeding.7
Dispersal strategy	Aquatic passive	Dispersal.1		Parasite	Feeding.8
	Aquatic active	Dispersal.2	Substrate preference	Coarse substrates	Substrate.1
	Aerial passive	Dispersal.3		Gravel	Substrate.2
	Aerial active	Dispersal.4		Sand	Substrate.3
Resistance form	Eggs/statoblasts	Resistance.1		Silt	Substrate.4
	Cocoons	Resistance.2		Macrophytes	Substrate.5
	Housings against desiccation	Resistance.3		Microphytes	Substrate.6
	Diapause/dormancy	Resistance.4		Twigs/roots	Substrate.7
	None	Resistance.5		Organic detritus	Substrate.8
Respiration method	Tegument	Respiration.1		Mud	Substrate.9
	Gill	Respiration.2	Velocity preference	Null	Velocity.1
	Plastron	Respiration.3		Slow	Velocity.2
	Spiracle	Respiration.4		Medium	Velocity.3
	Hydrostatic vesicle	Respiration.5		Fast	Velocity.4

### Statistical Analysis

Prior to analysis, macroinvertebrate community abundances were ln(x + 1) transformed to ensure consistent comparability with functional trait responses (see step iv in the trait processing procedure above). All analyses were performed using R version 3.0.2 (R Development Core Team 2014). The multivariate composition of macroinvertebrate community abundances (taxonomic) and functional traits comprising control and restored reaches was examined *via* a “Principal Coordinate Analysis” (PCoA) using a Bray–Curtis dissimilarity index, which was obtained using the “*cmdscale*” function in the “Vegan” package (Oksanen et al. 2016). Differences in the multivariate location (the position of the community centroid) between all samples from control and restored reaches were statistically tested *via* a nested “Permutational Multivariate Analysis of Variance” (PERMANOVA—using the “*adonis*” function; each paired control and restored site was used as a blocking factor in Vegan). The multivariate dispersion of macroinvertebrate communities residing in control and restored reaches was quantified by a “Permutational Analysis of Multivariate Dispersion” (PERMDISP—using the “*betadisper*” function in the Vegan package). These two sets of analyses were performed on both taxonomic and functional trait compositions. The significance of PERMDISP was determined *via* an analysis of variance (ANOVA). PERMANOVA and PERMDISP analyses were also repeated on samples only collected from organic biotopes (i.e., excluding macroinvertebrate samples taken from any mineralogical patches) due to the widespread occurrence of these biotopes across both control and restored reaches (see Results). The differences of individual taxa and traits (univariate responses) between control and restored reaches were also examined by performing “Similarity Percentages” (SIMPER) analysis *via* the “simper” function in Vegan. Taxa exclusively sampled from either control or restored reaches were assessed in terms of their numerical abundance (with those comprising <1% of the entire community population being classified as “rare”—*sensu* Ledger et al. 2009) and the number of samples that they were located within.

Statistical differences in macroinvertebrate compositions between biotopes were examined *via* a nested PERMANOVA, with each respective pair of control and restored reaches being used as a blocking factor. The alpha-diversity of faunal assemblages supported by each biotope was assessed for both taxonomic and functional trait compositions by calculating the inverse Simpson’s diversity (see Oksanen 2016), which was used to account for the fixed number of traits and their lack of independence (Larsen and Ormerod 2010). The beta-diversity of taxonomic and functional trait compositions within each biotope was quantified by calculating the multivariate dispersion (see Anderson et al. 2006) *via* PERMDISP to indicate the degree of ecological heterogeneity. Graphics for these diversity-biotope associations were prepared using the “ggplot2” package (Wickham and Chang 2016). Linear models were constructed between alpha- and beta-diversity values (dependent variables) and biotopes (independent variable) for both taxonomic and functional trait compositions, whereby model residuals were plotted against fixed values to assess the homogeneity of variances and Quantile-Quantile plots were inspected to ensure that models were normally distributed. Subsequently, a one-way ANOVA was performed on each of these models, with a “Tukey’s Honest Significant Difference” (THSD) post-hoc comparisons test being performed on those identified as significant (*α* = 0.05). This allowed pair combinations of biotopes supporting significantly different alpha- and beta-diversity values to be identified. Finally, a group-equalized “Indicator Value” (IndVal) analysis was conducted *via* the “multipatt’”function in the ‘indicspecies’ package (De Caceres and Jansen 2015) to examine the preferences of specific taxa and traits towards different biotopes and performed across 999 permutations to determine its significance.

## Results

### Macroinvertebrate Responses to Restoration Works

**Fig. 2:**
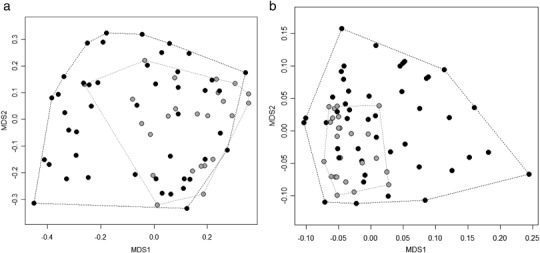
PCoA plots of macroinvertebrates communities between control and restored reaches for **a** taxonomic and **b** functional trait compositions. *Grey circles* = control reaches and *black circles* = restored reaches. A convex hull has been drawn to highlight differences in the area of multivariate space occupied by each factor

PERMANOVA highlighted that control and restored reaches possessed significantly different multivariate locations for both taxonomic (*F* = 4.05, *p* = ≤0.001) and functional trait compositions (*F* = 5.17, *p* ≤ 0.001), but this only accounted for 6% (*r* ^2^ = 0.06) and 7% (*r* ^2^ = 0.07) of the statistical variance, respectively. PERMDISP demonstrated that the amount of multivariate dispersion differed significantly between samples from control and restored reaches for both taxonomic (*F* = 13.96, *p* ≤ 0.001) and functional trait compositions (*F* = 16.71, *p* ≤ 0.001) and accounted for 17% (*r* ^2^ = 0.17) and 20% (*r* ^2^ = 0.20) of the statistical variance, respectively. PCoA plots highlighted subtle shifts in the community centroid between control and restored reaches, with samples from the latter clearly possessing higher levels of multivariate dispersion, most evidently for functional trait compositions (Fig. 2). Analyses conducted on samples taken exclusively from organic biotopes (i.e., with those from mineralogical biotopes being excluded) highlighted that neither the multivariate location or dispersion differed significantly between control and restored reaches for both taxonomic (PERMANOVA: *F* = 0.93, *r* ^2^ = 0.02, *p* = 0.376; PERMDISP: *F* = 0.58, *r* ^2^ = 0.01, *p* = 0.449) and functional trait compositions (PERMANOVA: *F* = 1.98, *r* ^2^ = 0.04, *p* = 0.054; PERMDISP: *F* = 0.95, *r* ^2^ = 0.02, *p* = 0.334). SIMPER analysis highlighted 9 macroinvertebrate families (spanning various taxonomic orders) and seven traits (from various grouping features) differed significantly between control and restored reaches (see Table 2). All of these were higher on average at control sites, with the exception of the trait “substrate.3” (community preferences towards sand substrates) which increased on average within restored reaches. Some taxa were unique to restored reaches (Caenidae, Ceratopogonidae, Corixidae, Hydrophilidae, Pediciidae and Tipulidae), while others were only sampled within control sites (Muscidae, Naucoridae, Planariidae, Psychomyiidae); but all of these taxa were numerically rare and were only recorded in one sample except for Caenidae (seven samples; Order: Ephemeroptera) and Tipulidae (two samples; Order: Diptera).

**Table 2:** Mean average (±1 standard deviation) (a) taxa abundances and (b) trait values in control and restored reaches which differed significantly based on SIMPER analysis

	Taxa/Trait	Control	Restored	*p*-value
(a)	Asellidae	37.20 (58.44)	28.70 (54.59)	0.001***
	Crangonyctidae	0.73 (1.72)	0.63 (1.56)	0.025*
	Gammaridae	11.84 (20.47)	9.43 (15.62)	0.014*
	Glossiphoniidae	1.25 (2.75)	0.95 (2.6)	0.047*
	Muscidae	0.02 (0.14)	0.02 (0.12)	0.012*
	Naucoridae	0.02 (0.14)	0.02 (0.12)	0.012*
	Planariidae	0.02 (0.14)	0.02 (0.12)	0.02*
	Polycentropodidae	1.08 (2.65)	1.14 (2.59)	0.044*
	Psychomyiidae	0.02 (0.14)	0.02 (0.12)	0.013*
(b)	Feeding.group.3	0.36 (0.015)	0.23 (0.020)	0.002**
	Feeding.group.8	0.02 (0.003)	0.02 (0.001)	0.042*
	Food.3	0.18 (0.007)	0.12 (0.009)	0.003**
	Food.9	0.02 (0.004)	0.01 (0.002)	0.029*
	Reproduction.1	0.42 (0.013)	0.29 (0.02)	0.049*
	Substrate.3	0.08 (0.004)	0.11 (0.005)	0.036*
	Substrate.6	0.07 (0.003)	0.05 (0.003)	0.007**

*p ≤ 0.05; **p ≤ 0.01; ***p ≤ 0.001

### Differences in Macroinvertebrate Compositions Between Biotopes

**Fig. 3:**
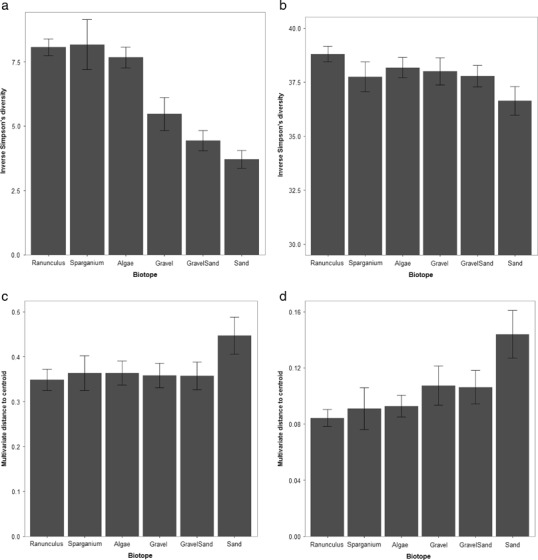
Alpha-diversity (Inverse Simpson’s) and beta-diversity (multivariate dispersion) measures of macroinvertebrate taxonomic and functional trait compositions across different biotopes **a** Inverse Simpson’s diversity measure for taxonomic compositions; **b** Inverse Simpson’s diversity measure for trait compositions; **c** Multivariate dispersion of taxonomic compositions; **d** Multivariate dispersion of trait compositions

Control reaches were dominated by organic biotopes in all instances, so that bare patches of mineral substrates were not visible from the river bank (and subsequently not sampled). Restored reaches almost always comprised the same organic biotopes as their respective control reach and additionally included distinct patches of bare mineralogical substrates (as evident from aerial imagery—see Supplementary Material). PERMANOVA highlighted the average community composition values for both taxonomic (*F* = 4.19, *p* ≤ 0.001) and functional trait compositions (*F* = 4.54, *p* ≤ 0.001) differed significantly between dominant biotopes, which explained 25% (*r* ^2^ = 0.25) and 26% (*r* ^2^ = 0.26) of the total statistical variance, respectively. The alpha-diversity of macroinvertebrate taxonomic compositions was typically higher within organic habitats (Fig. 3a) and differed significantly between biotopes (ANOVA: *F* = 12.62, *p* ≤ 0.001), accounting for 50% of the statistical variation (*r* ^2^ = 0.50). This pattern was less pronounced when functional trait responses were considered (Fig. 3b), with no significant difference occurring between the inverse Simpson’s diversity value for different biotopes (ANOVA: *F* = 1.78, *r* ^2^ = 0.12, *p* = 0.129). Sand exhibited the lowest average alpha-diversity values for both forms of biotic information (Fig. 3). THSD highlighted significant differences between the alpha-diversity of taxonomic compositions for all pair combinations of biotopes comprising one organic and one mineralogical habitat. The most highly significant values recorded occurred where alpha-diversity values from different organic biotopes were compared against finer substrates: gravel (*p* = 0.007–0.036), gravel and sand (*p* = ≤0.001–0.003) and sand (*p* = ≤0.001). Conversely, beta-diversity was highest in sand for both taxonomic (Fig. 3c) and functional trait compositions (Fig. 3d). However, the former displayed comparable degrees of multivariate dispersion between biotopes (accounting for 8% of the statistical variance−*r* ^2^ = 0.08) and did not differ significantly (ANOVA: *F* = 1.16, *p* = 0.338), compared to the latter which differed significantly (ANOVA: *F* = 3.61, *p* = 0.006) and explained 22% of the statistical variance (*r* ^2^ = 0.22). THSD demonstrated that the beta-diversity of functional trait compositions within sand samples differed significantly from *Ranunculus*. sp (*p* = 0.002), *S. Emersum* (*p* = 0.035) and macroalgae (*p* = 0.013), while all other pairwise comparisons of biotopes were not significant. IndVal analysis indicated eight macroinvertebrate families and traits were associated with specific combinations of biotopes (see Table 3). Organic biotopes were associated with a higher number of ecological responses compared to mineralogical patches. *S. emersum* was the only individual biotope found to be associated with a specific macroinvertebrate response (“Respiration.3”—the trait denoting fauna possessing plastron respiration) and this macrophyte was present within six out of seven biotope combinations comprising significant ecological preferences. Sand was associated with the lowest number of macroinvertebrate taxa and traits. None of the taxa unique to control or restored reaches were significantly associated with specific biotopes. Caenidae (Order: Ephemeroptera) and Tipulidae (Order: Diptera the two taxa that were unique to restored reaches and found in more than one sample) were collected from one and zero patches of bare substrate, respectively.

**Table 3:** Macroinvertebrate taxa (a) and traits (b) significantly associated with different biotopes based on IndVal analysis

Biotopes.	Ecological inference	Taxa	Indicator value	*p*-value	Traits	Indicator value	*p*-value
*S. emersum*	Prefers S. Emersum (and potentially other broad-leaved macrophytes).	–	–	–	Respiration.3	0.55	0.020*
*Ranunculus* sp. and *S. emersum*	Prefers different types of submerged macrophytes.	Crangonyctidae	0.65	0.005**	Reproduction.6	0.62	0.028*
*Ranunculus* sp., *S. emersum* and algae	Prefers various types of organic biotopes.	Polycentropodidae	0.62	0.018*	Feeding.6	0.79	0.002**
					Food.9	0.75	0.003**
*Ranunculus* sp., *S. emersum* and Gravel	Prefers different types of macrophytes and coarse substrates.	Simuliidae	0.76	0.003**	–	–	–
Macroalgae, Gravel, Gravel and sand, Sand	Repudiates different types of macrophytes.	–	–	–	Feeding.1	0.85	0.019*
					Resistance.2	0.85	0.023*
*Ranunculus* sp., *S. emersum*, Macroalgae and Gravel	Repudiates the presence of sand (and potentially other types of fine sediment).	Gammaridae	0.87	0.003**	Respiration.4	0.77	0.040*
		Glossiphoniidae	0.71	0.018*			
		Hydropsychidae	0.67	0.015*			
*Ranunculus* sp., *S. emersum*, Macroalgae, Gravel, Gravel & sand	Repudiates pure sand (and potentially other types of fine sediment).	Asellidae	0.92	0.001***	–	–	–
		Baetidae	0.90	0.009**			
*S. emersum*, Macroalgae, Gravel, Gravel & sand and Sand	Repudiates Ranunculus (and potentially other fine-leave macrophytes).	–	–	–	Size.1	0.85	0.026*

*p ≤ 0.05; **p ≤ 0.01; ***p ≤ 0.001

## Discussion

### Ecological Responses to River Restoration Projects

River restoration projects often involve physically altering channel morphologies (channel shape, size and configuration) to enhance the quality and quantity of instream habitats. This is evident in the UK where the national “River Restoration Centre” has compiled a handbook of restoration techniques which outlines various methods and case studies on morphologically rehabilitating rivers subject to a range of environmental conditions and constraints (River Restoration Centre 2013). This study examined three restoration projects which aimed to reinstate a pre-disturbed braided planform by creating a multi-channel system. Such techniques are novel within the UK, as efforts have been historically focused on reworking the structure of a single channel. As such, the restoration projects examined within this study differ drastically from others across the UK which have been the subject of globally recognized post-project appraisals (e.g., Biggs et al. 1998; Pretty et al. 2003; Harrison et al. 2004).

This study found that several macroinvertebrate taxa displayed a reduction in occurrence within restored reaches, specifically the crustaceans Asellidae and Gammaridae. This could be attributed to a reduced amount of coarse organic particulate matter being retained within mineralogical patches substrates, as the proportion of the ‘shredders’ also declined within restored reaches. This is in contrast to the findings of Jähnig et al. (2009), who reported that the same feeding group displayed the opposite trend between paired single-channel and multi-channel river sections.

The multivariate location (i.e., the community centroid) of macroinvertebrate community abundances (taxonomic) and functional trait compositions differed marginally between control and restored reaches. Differences in the position of community centroids typically suggests contrasting biotic assemblages (see Boersma et al. 2016), but such patterns accounted for a low amount of statistical variation in the present study and univariate analyses found limited evidence to indicate a structural or functional turnover of macroinvertebrate communities (see below). As such, it is unlikely that changes in the average community composition were due to control and restored reaches exhibiting contrasting taxonomic and functional trait compositions. Modest biotic responses to river restoration practices have been widely reported in many post-project appraisals (see Roni et al. 2008; Miller et al. 2010; Palmer et al. 2010). Such patterns may occur if the diversity and quality of biotopes does not represent the primary limiting factor constraining biotic communities (Palmer et al. 2005, 2010) and large-scale environmental pressures persist (Poff 1997). As such, high levels of heavy metals and nutrient pollution or flow regime modifications within the Tame catchment (Lawler et al. 2006; Langford et al. 2010) may have limited macroinvertebrate community responses to restoration activities. Given the low amount of statistical variation accounted for by changes in the multivariate location, the evident contrast in the amount of multivariate dispersion between control and restored reaches was probably a key factor driving the significant difference in the average community composition recorded (see Anderson and Walsh 2013).

The greater degree of multivariate dispersion (ecological heterogeneity) in restored reaches for both taxonomic and functional trait compositions relative to control sites suggests that restoration works increased the heterogeneity of macroinvertebrate communities. Such responses may have arisen if restored reaches promoted the colonization of more functionally heterogeneous taxa (Houseman et al. 2008; Boersma et al. 2016). However, fauna sampled exclusively within restored reaches comprised a very small percentage of the total community abundance and in most instances were only found in a single sample (with two exceptions, see below). As such, the increase in the multivariate dispersion of biotic communities at restored reaches was probably due to differences in small-scale environmental heterogeneity which provided local ecological benefits, such as the presence of distinct habitat patches (Heino et al. 2012).

### Taxonomic and Functional Trait Compositions Across Different Biotopes

This study found that sampling macroinvertebrate communities from distinct lotic habitats elucidated detailed ecological responses to river restoration practices, as reported in previous studies (e.g., Jähnig and Lorenz 2008; Verdonschot et al. 2016). While taxa would have been likely to drift between control and restored reaches, sampling from distinct biotopes in this study demonstrated that macroinvertebrates did not widely establish within novel habitats supported by rehabilitated sections of river. For example, those taxa sampled exclusively from restored reaches were numerically rare and recorded within a maximum of one sample from a mineralogical biotope, thus highlighting that the reinstatement of these novel habitats did not facilitate the widespread (re)colonization of “new” taxa. Biotopes have been found to support distinct macroinvertebrate taxonomic compositions in numerous studies (e.g., Armitage et al. 1995; Harper et al. 1998; Buffagni et al. 2000), but few have quantified their functional properties associated with distinct riverine biotopes (but see Demars et al. 2012). This study found that a range of taxa and functional traits were significantly associated with different combinations of biotopes. For example, *Sparganium emersum* (a broad-leaved macrophyte) was associated with fauna respiring through permanent air stores (plastrons), which often reside in the upper parts of the water column (Chapman et al. 2004). This suggests that the ecology of *S. emersum*, which typically extends from the riverbed through the water column to the surface, provides a niche habitat for taxa possessing this respiratory trait. In addition, the two dominant macrophytes recorded in this study, *S. emersum* and *Ranunculus* sp. (a fine-leaved macrophyte) were significantly associated with taxa that reproduce by depositing groups of eggs within vegetation. These biotopes were also significantly associated with the non-native amphipod Crangonyctidae, which is in keeping with the findings of Macneil and Dick (2014), who found that this taxon was positively associated with macrophyte cover.

This study highlighted that macroinvertebrate communities varied significantly between biotopes (which accounted for a higher amount of statistical variation than the influence of restoration alone) and indicated that they support different ecological functions, as reported in previous studies (e.g., Harper et al. 1992; Storey and Lynas 2007; Demars et al. 2012). The alpha-diversity of taxonomic compositions was higher in organic biotopes relative to mineralogical habitats, although this was less clear for functional traits (see below). Organic biotopes probably supported a greater number of taxa due to the array of ecological functions provided by such habitats, including them providing a refuge from predators or a platform from which macroinvertebrates can consume detritus (Harper and Everard 1998; Wharton et al. 2006). Sand supported the lowest alpha-diversity measures, as commonly reported within previous studies (e.g., Wood 1998; Larsen and Ormerod 2010; Demars et al. 2012). However, sand exhibited the highest beta-diversity (multivariate dispersion) relative to other biotopes, indicating greater ecological heterogeneity (particularly for the functional properties of communities) existed among individual patches of sand, even if the alpha-diversity within each sample was low.

The identification of biotopes which can support distinct and diverse biotic assemblages may help guide the management and conservation of key habitats during restoration works; this has been advocated as the first step of implementing ecologically effective river restoration strategies (see Roni et al. 2008). This study highlighted that organic biotopes supported the greatest alpha diversity and should be preserved when conducting future management schemes along the studied watercourse. In addition, sampling dominant biotopes provided a basis for understanding biotic responses to river restoration practices by demonstrating that greater ecological heterogeneity existed between mineralogical biotopes (most notably sand). This corroborated the higher amount of multivariate dispersion displayed within restored reaches, which broadly concurs with the findings of Jähnig and Lorenz (2008). Further evidence of this within the present study is provided by the multivariate dispersion not differing significantly between control and restored reaches when only organic biotopes were considered. As such, this study demonstrates the advantages of examining the biotic compositions supported by distinct biotopes as a basis for understanding ecological responses to restoration projects and could be used within future post-project appraisals to better inform management practices.

### Utilizing Functional Traits in Post-Project Appraisals

Functional responses of biotic communities have not been frequently reported within river restoration post-project appraisals (Kail et al. 2015). Such community characteristics could potentially facilitate a priori predictions of ecological responses to river restoration practices (e.g., Lamouroux et al. 2015; Dolédec et al. 2015) and potentially pinpoint where the reinstatement of habitats may yield maximum functionality and biodiversity gains. For example, Verdonschot et al. (2016) found that a functional trait metric based on habitat preferences was more intrinsically linked to habitat heterogeneity induced by river restoration works and attributed this to the creation of specific biotopes (e.g., cobbles and fine particulate organic matter). In addition, Tullos et al. (2009) reported that rehabilitation projects acted as a disturbance, with restored reaches supporting taxa which were resistant or resilient to the physical disruption of the riverbed associated with restoration activities, including multivoltinism and high fecundity.

The utilization of functional traits has been limited within the UK thus far (notable exceptions being Demars et al. 2012; White et al. 2017) and their use has been cautioned by some authors due to their potential sensitivity to extraneous factors, such as the inter-correlation between traits (Poff et al. 2006) or the overriding influence of the most abundant taxa (precluding mechanistic ecological associations—Pilière et al. 2016). Indeed, Tomanova and Usseglio-Polatera (2007) reported that some individual trait responses to environmental variables were difficult to interpret and we experienced similar difficulties in this study, such as taxa that consume vertebrates being significantly associated with organic biotopes. However, numerous trait associations within this study displayed obvious ecological expression, such as macrophytes supporting fauna reproducing by laying groups of eggs in vegetation and taxa displaying an affinity with sand substrates increasing at restored sites (where this biotope widely occurred). This study found that the alpha-diversity of macroinvertebrate functional trait compositions differed less profoundly between organic and mineralogical biotopes relative to taxonomic compositions. This suggests a redundancy of functional traits within organic biotopes, whereby increasing taxonomic diversity will not result in a higher functional diversity and that mineralogical biotopes support a smaller number of taxa capable of performing the same number of functions (Bêche and Resh 2007; Larsen and Ormerod 2010). However, in this study functional trait compositions were more sensitive to multivariate dispersion measures (beta-diversity) compared to taxonomic communities, particularly when considering the difference between biotopes.

In recognition of the need to consider community responses to different environment controls, multivariate analyses have been advocated within the context of restoration ecology (Matthews and Spyreas 2010; Boersma et al. 2016). Such analytical techniques have also been encouraged for analyzing aquatic macroinvertebrate functional traits (Poff et al. 2006) and such approaches have been found to yield comparable statistical outcomes across varying taxonomic resolutions (e.g., Gayraud et al. 2003; Demars et al. 2012). Multivariate dispersion was found to be a key measure of ecological responses in this study as it allowed the heterogeneity (beta-diversity) of macroinvertebrate communities to be quantified (Anderson et al. 2006). Previous studies examining the multivariate dispersion of ecological communities have found that similar results can be obtained using family-level data and/or species-level data (e.g., Terlizzi et al. 2009; Hill et al. 2016), and that finer taxonomic resolutions may not necessarily be required to guide management strategies. It is important that future post-project appraisals should examine the multivariate dispersion alongside average differences in community compositions (i.e., multivariate location—see Anderson and Walsh 2013), as it can be a valuable indicator of ecological responses to changes in habitat compositions (e.g., Heino et al. 2012). Such analyses have rarely been explored within the context of functional traits (see Schmera et al. 2017), but results from this study highlight that the multivariate dispersion of functional traits should be examined within future river restoration post-project appraisals.

## Electronic supplementary material

Supplementary Material
