# Supplementary material for: Macroinvertebrate Taxonomic and Functional Trait Compositions within Lotic Habitats Affected By River Restoration Practices
Source: Environ Manage. 2017 May 17;60(3):513–25. doi: 10.1007/s00267-017-0889-1 (PMC5544791; doi:10.1007/s00267-017-0889-1)
Supplement: Supplementary file 1 — Supplementary Material [file 267_2017_889_MOESM1_ESM.docx]

**Supplementary Material**

Appendix A.

The following appendix contains details of the three restoration projects examined in this study along the River Tame. All projects involved widening channel outlines by removing large sections of one bank. The remnants of the initial bank were then fashioned as islands which diverted flows into multiple separate channels (mimicking a braided channel form). All restoration projects were paired with a nearby non-restored (control) site. One project was conducted in northeast Birmingham (West Midlands, UK) close to the village of ‘Minworth’ (the site name for this project; see Figure A1). The remaining two projects were conducted along adjacent river reaches (see Figure A1) at the ‘Middleton Lakes’ site managed by the ‘Royal Society for the Protection of Birds’ (RSPB). The first project (Middleton Lakes A) was conducted in 1998, while restoration works for the second project (Middleton Lakes B) were carried out in 2006. The physico-chemical properties of each site were obtained from nearby water quality stations were obtained from the ‘Water Quality Archive’ (available through: <http://environment.data.gov.uk/water-quality/view/landing>) managed by the Environment Agency (the statutory environmental regulator within the UK).

Figure A1. Study sites across the River Tame, with the site names displayed. ML = Middleton Lakes; D/S Minworth STW = Downstream of Minworth Sewages Treatment works; square (within inset) = Study location; triangles = control sites and circles = restored sites; squares = water quality stations.


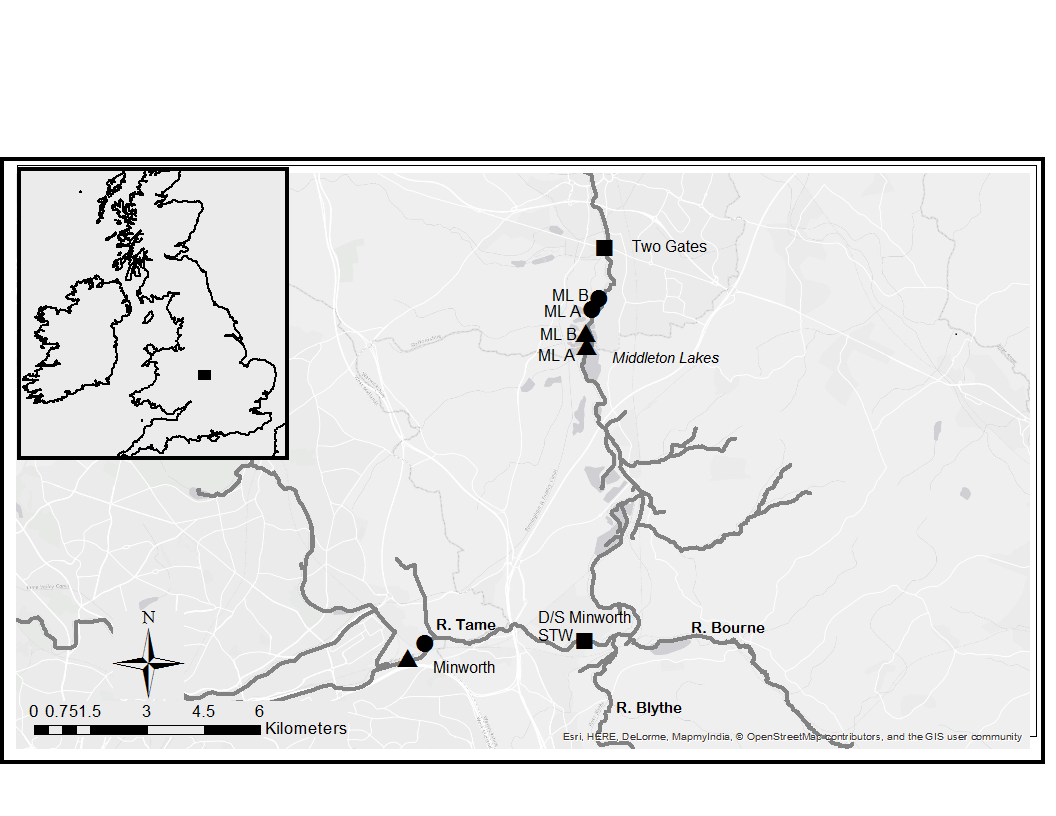


Table A1. Physico-chemical properties of the restoration projects.

| **Water quality parameter** | **Minworth^a^** | **Middleton Lakes^b^** |
| --- | --- | --- |
| pH | 7.54 | 8.07 |
| Orthophosphate (mg/l) | 0.33 | 0.42 |
| Nitrate (mg/l) | 10.7 | 11.4 |
| Oxygen (mg/l) | 6.62 | 8.11 |
| Oxygen (% saturation) | 69.3 | 84.8 |
| Nickel (filtered; µg/l) | 14.1 | 11.7 |
| Zinc (filtered; µg/l) | 38.4 | 20 |

^a^ River Tame - D/S Minworth STW bacteria beds on 13/08/2014.

^b^ River Tame – Two Gates, Fazely on 02/09/2014.

|  | **Minworth** | **Middleton Lakes A** | **Middleton Lakes B** |
| --- | --- | --- | --- |
| Date of completion | 2008 | 1998 | 2006 |
| Reach length (m) | 384 | 253 | 500 |
| Width increase (m) | 28.5 | 21 | 27.5 |
| Number of mature islands created | 3 | 10 | 28 |
| Number of mid-channel bars created | 0 | 0 | 10 |

Table A2. The project details of the restoration works.

Restoration works at Minworth were conducted as part of a flood alleviation scheme and project works involved increasing the average channel width from 13 to 41.5 metres. Widening the channel led to the widespread deposition of bare mineralogical substrates – gravel and sand patches (particularly along the west bank - see Figure A2a) relative to the control site (see Figure A2b).

Figure A2. Aerial imagery of the a) restored (source: Google Earth, 2013a) and b) control (source: Google Earth, 2013b) reaches at Minworth.

| 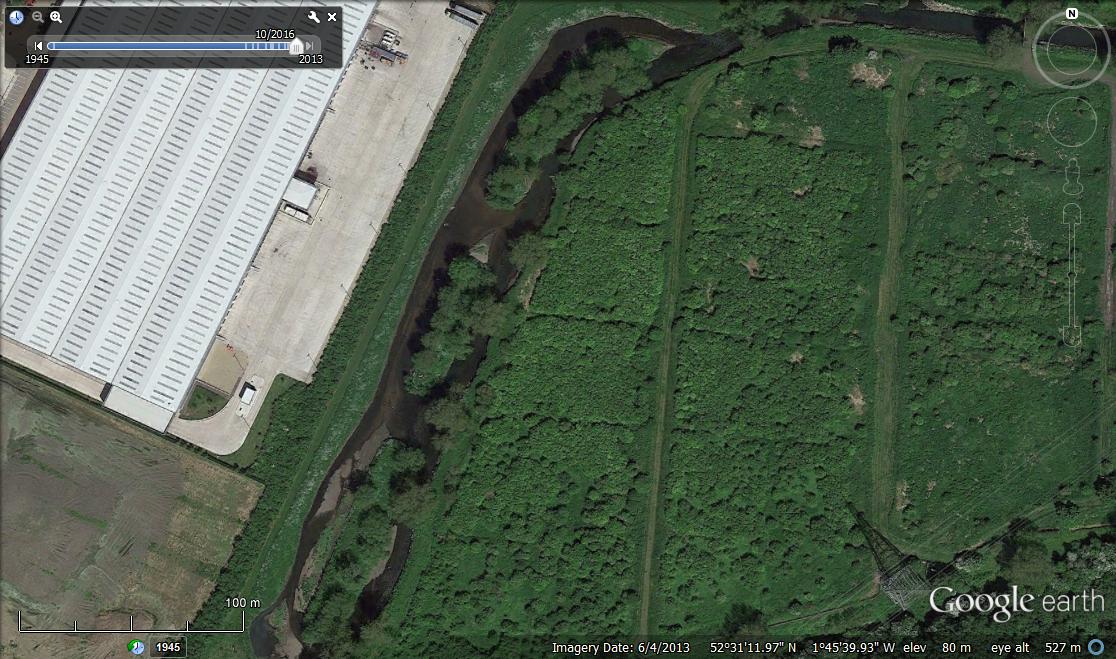 **N**  **100m** | 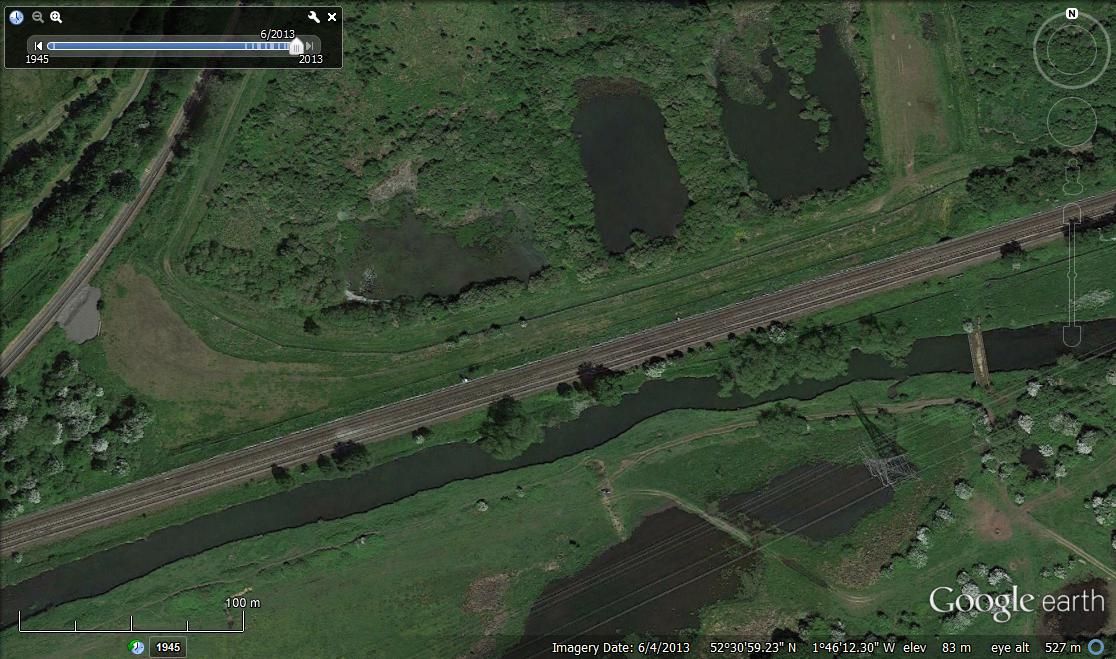  **100m**  **N** |
| --- | --- |

Both restoration projects at Middleton Lakes were conducted as part of gravel extraction schemes. Works conducted for Middleton Lakes A were carried out in 1998 and the average channel width was increased from 20.5 to 41.5 metres. A number of vegetated islands were maintained in the centre of the channel (in the position of the original eastern bank) and there were evident coverings of bare substrates (particularly along the eastern bank – see Figure A3a) relative to the control sites (see Figure A3b). The restoration project at Middleton Lakes B was conducted eight years later (2006), with the average channel width being increased from 21 to 48.5 metres. However, the design of this restoration project also incorporated fashioning mid-channel bars towards the north-eastern bank which become inundated at higher flows (see Figure A3a). The deposition of bare mineralogical substrates was also evident across large parts of the restored reach.

Figure A3. Aerial imagery of the a) restored (source: Google Earth, 2013c) and b) control (source: Google Earth, 2013d) reaches at the Middleton Lakes restoration sites. Red arrow = Middleton Lakes A, blue arrow = Middleton Lakes B.

a)

**
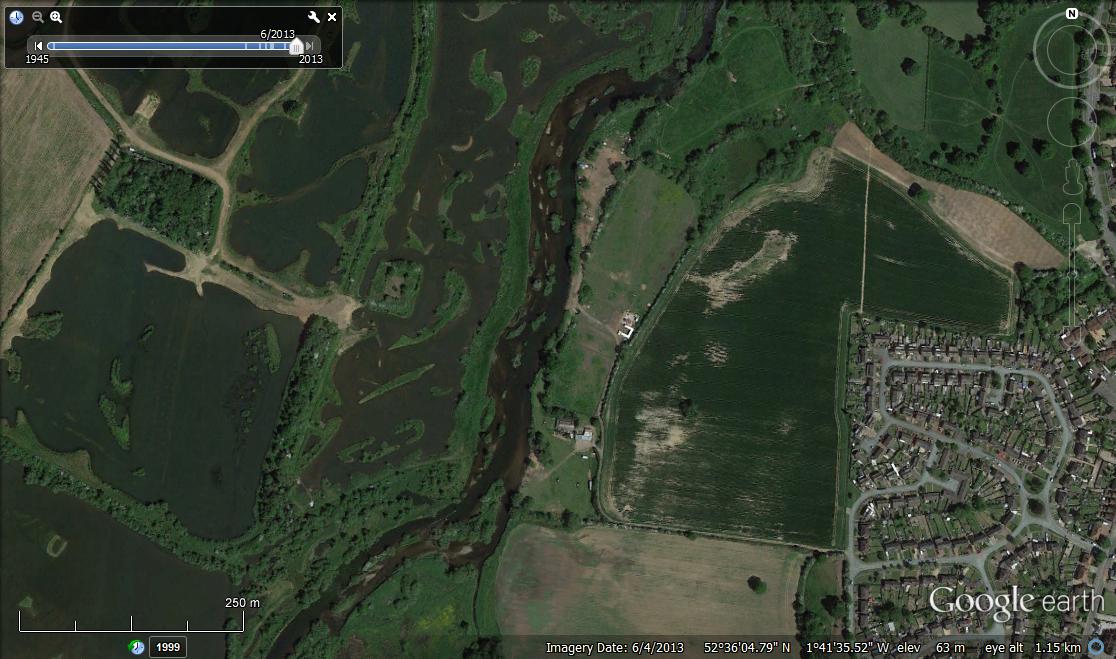
**

**250m**


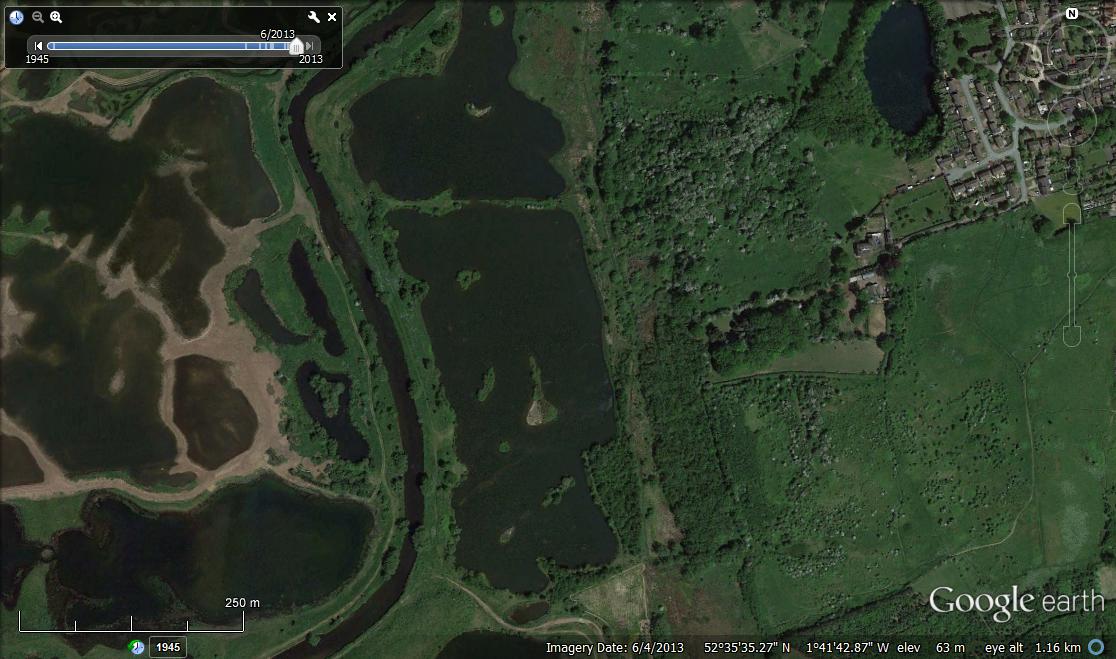
b)

**250m**

**References**

Google Earth (2013a) V 7.1.5.1557 (6/4/2013) Birmingham, UK. 52°31’11.97”N; 1°45’39.93”W. Eye altitude 527m. Software available through: <http://www.earth.google.com> [Accessed 11/10/2016 – citation referring to Figure A2a].

Google Earth (2013b) V 7.1.5.1557 (6/4/2013) Birmingham, UK. 52°30’59.23”N; 1°46’12.30”W. Eye altitude 527m. Software available through: <http://www.earth.google.com> [Accessed 11/10/2016 – citation referring to Figure A2b].

Google Earth (2013c) V 7.1.5.1557 (6/4/2013) Tamworth, UK. 52°36’04.79”N; 1°41’35.52”W. Eye altitude 1.15km. Software available through: <http://www.earth.google.com> [Accessed 11/10/2016 – citation referring to Figure A3a].

Google Earth (2013d) V 7.1.5.1557 (6/4/2013) Tamworth, UK. 52°35’35.27”N; 1°41’42.87”W. Eye altitude 1.16km. Software available through: <http://www.earth.google.com> [Accessed 11/10/2016 – citation referring to Figure A3b].
